# Regulation of Neuronal Morphogenesis and Positioning by Ubiquitin-Specific Proteases in the Cerebellum

**DOI:** 10.1371/journal.pone.0117076

**Published:** 2015-01-21

**Authors:** Julius Anckar, Azad Bonni

**Affiliations:** 1 Department of Neurobiology, Harvard Medical School, Boston, Massachusetts, United States of America; 2 Department of Anatomy and Neurobiology, Washington University School of Medicine, St Louis, Missouri, United States of America; Institut de la Vision, FRANCE

## Abstract

Ubiquitin signaling mechanisms play fundamental roles in the cell-intrinsic control of neuronal morphogenesis and connectivity in the brain. However, whereas specific ubiquitin ligases have been implicated in key steps of neural circuit assembly, the roles of ubiquitin-specific proteases (USPs) in the establishment of neuronal connectivity have remained unexplored. Here, we report a comprehensive analysis of USP family members in granule neuron morphogenesis and positioning in the rodent cerebellum. We identify a set of 32 USPs that are expressed in granule neurons. We also characterize the subcellular localization of the 32 USPs in granule neurons using a library of expression plasmids encoding GFP-USPs. In RNAi screens of the 32 neuronally expressed USPs, we uncover novel functions for USP1, USP4, and USP20 in the morphogenesis of granule neuron dendrites and axons and we identify a requirement for USP30 and USP33 in granule neuron migration in the rodent cerebellar cortex *in vivo*. These studies reveal that specific USPs with distinct spatial localizations harbor key functions in the control of neuronal morphogenesis and positioning in the mammalian cerebellum, with important implications for our understanding of the cell-intrinsic mechanisms that govern neural circuit assembly in the brain.

## Introduction

Control of neuronal positioning and morphogenesis is essential for the establishment of neuronal connectivity in the developing brain. In the cerebellar cortex, newly generated granule neurons integrate into neural circuits after a stereotyped set of fundamental developmental events [1], [2]. Upon their generation in the external granule layer (EGL), granule neurons undergo polarization and axon growth. The growth of granule neuron parallel fiber axons continues even as granule neuron somas migrate through the molecular layer and past Purkinje cells into the internal granule layer (IGL) [3], [4]. Once in the IGL, granule neurons undergo dendrite differentiation and complete their maturation, leading to the establishment of neuronal connectivity with afferent neurons in the IGL and efferent neurons in the molecular layer [5–7].

In recent years, cell-intrinsic mechanisms that control distinct phases of granule neuron morphogenesis and connectivity in the cerebellum have been identified (for reviews, see [8], [9]). Among these mechanisms, ubiquitin signaling pathways have emerged as key regulators of granule neuron morphogenesis and connectivity [10]—[14]. These studies have revealed that spatial localization of E3 ubiquitin ligases plays a critical role in distinct aspects of granule neuron development, a principle that also holds in the control of neuronal morphogenesis and connectivity in other regions of the nervous system [9]. Although the functions of E3 ubiquitin ligases in neuronal development are beginning to be elucidated [9], [15], the roles of other classes of ubiquitin pathway enzymes in neuronal development remain poorly understood.

Ubiquitination, the covalent attachment of a single ubiquitin moiety or ubiquitin chains to cellular proteins, is a key modification that regulates fundamental cellular processes. Ubiquitination often leads to proteasomal degradation of substrates, but also controls protein localization, activity, and complex assembly [16]. Ubiquitin attachment is mediated by successive enzymatic steps executed by a single ubiquitin E1 enzyme, one of ~50 ubiquitin E2 conjugating enzymes and one of ~500 of ubiquitin E3 ligases. The complexity of ubiquitination events is further increased by the action of deubiquitinating enzymes (DUBs), which maintain cellular levels of free ubiquitin and remove ubiquitin from substrates and thus counteract the activity of the ubiquitin-conjugating machinery. The roles of DUBs in neuronal morphogenesis and connectivity have remained largely unexplored.

The mammalian genome contains an estimated 90 DUB genes, many of which show tissue-specific expression pattern [17]. Based on the architecture of the ubiquitin protease domain, DUBs are grouped into five categories: the ubiquitin-specific proteases (USPs), the ubiquitin C- terminal hydrolases (UCHs), the Josephins, the ovarian tumor proteases (OTUs), and the JAB1/MPN/MOV34 metalloenzymes (JAMMs). The large number of DUB members suggests that individual members might have specialized properties and function in distinct subcellular locales. Among the DUBs, the USP family is by far the largest, comprising roughly 50 members, although some USP members lack deubiquitinating activity [17]. In addition to a highly conserved catalytic domain, USPs are rich in N- and C-terminal extensions that contain targeting sequences or protein-protein interaction motifs. USPs often harbor catalytic activity toward multiple types of ubiquitin chains, suggesting that the deubiquitinating activity of USPs might be directed toward specific protein substrates or operate at distinct subcellular locations [18], [19]. Hence, elucidation of the signaling pathways controlled by USPs is likely to be of considerable importance for understanding specific ubiquitin-mediated biological functions.

To illuminate the unexplored functions of deubiquitinating enzymes in mammalian brain development, we have carried out a comprehensive analysis of the ubiquitin-specific proteases (USPs) in granule neuron morphogenesis and positioning in the rodent cerebellum. We have identified 32 USPs that are expressed in granule neurons. Using a library of expression plasmids encoding GFP-USPs, we have also characterized the subcellular localization of the 32 USPs in granule neurons. In RNAi screens of the 32 neuronally expressed USPs, we have uncovered novel functions for USP1, USP4, and USP20 in the morphogenesis of dendrites and axons in granule neurons. Importantly, in an RNAi screen of the 32 USPs in rat pups, we have discovered that USP30 and USP33 play critical roles in granule neuron migration in the rodent cerebellar cortex *in vivo*. These studies define novel functions for specific USPs in the control of granule neuron morphogenesis and positioning, and thus advance our understanding of the cell-intrinsic mechanisms governing neural circuit assembly in the mammalian cerebellum.

## Materials and Methods

### Ethics

All animal experiments were conducted under the institutional guidelines and were approved by the Institutional Animal Care and Use Committee (IACUC) of Harvard Medical School.

### Plasmids

Expression plasmids encoding GFP-tagged deubiquitinases (DUBs) were generated by PCR using cDNA from dissociated cultures of cerebellar granule neurons from rat pups. The protein coding region of each DUB was inserted into pEGFP-C1 or pEGFP-N1 vectors. cDNA for human USP2, USP16, USP25 and USP46 was kindly provided by J. Wade Harper (Harvard Medical School; [20]). For the construction of USP RNAi plasmids, oligonucleotides containing a 20–21 nucleotide target sequence were ligated into the pBS/U6 plasmid backbone [21]. The target sequences for the shRNA constructs is presented in S1 Table.

### Cell culture

Cerebellar granule neurons were isolated from P6 Long Evans rat pups and plated onto coverslips or tissue culture dishes coated with poly-L-ornithine as described [22]. For morphology experiments, granule neurons were transfected using a modified version of the calcium phosphate method as described [12]. 293T cells (ATCC) were cultured in DMEM supplemented with 10% calf serum, L-glutamine and antibiotics, and transfected using Lipofectamine 2000.

### Immunocytochemistry

Cerebellar granule neurons were fixed in 4% PFA and 4% sucrose for 10 minutes at room temperature, washed twice with PBS and permeabilized in PBS containing 0.4% Triton-X for 10 minutes. After a blocking step (8% milk, 1% goat serum in PBS), cells were stained in humidified staining chambers using the GFP (NeuroMab) or dsRED (Clonetech) antibody. After three washes with PBS, samples were stained with secondary, fluorophore-coupled antibodies and DNA stained with the DNA dye bisbenzimide (Hoechst). Coverslips were mounted on microscope slides using fluoromount G (SouthernBiotech) and analyzed on a NIKON eclipse TE2000 epifluorescence microscope as described [23].

### Quantitative real-time RT-PCR

Granule neuron RNA was extracted using Trizol reagent according to the manufacturer's instructions, and converted to cDNA using Superscript III (Invitrogen). 10 ng of cDNA was amplified by PCR using SYBRgreen as a fluorophore, and mRNA levels measured using the 2^-ddCT method using GAPDH as a reference housekeeping gene. The sequences of the primers are shown in S2 Table.

### *In vivo* electroporation

For RNAi experiments *in vivo*, the synapsin-promoter-mCitrin plasmid and RNAi plasmids were injected into the cerebellum of P4 rat pups using a custom-made 31gauge syringe (Hamilton). Electroporations were performed using a BTX ECM 830 Electro Square Porator (175V for 50ms, with 950ms intervals, 5 pulses), after which the pups were returned to their mother. At P9, pups were sacrificed and the cerebellum removed. GFP-positive cerebelli were identified using fluorescence microscopy, fixed for 1h on ice in PBS containing 4% PFA and 4% sucrose, and embedded in cryoprotectant (30% sucrose in PBS) overnight at 4 degrees. Next, cerebelli were embedded in OCT and flash frozen using liquid nitrogen, and frozen 40 uM coronal sections were prepared using a cryostat.

### Immunohistochemistry

Coronal sections were incubated in blocking solution (10% goat serum, 2% BSA, 0.5% Triton X-100 in PBS) for 1h at room temperature prior to incubation with primary antibodies diluted in blocking solution overnight at 4°C. The primary antibodies used were a rabbit anti-GFP (1:1000 dilution) and mouse anti-Calbindin (Sigma, 1:500 dilution). The sections were washed for 15 minutes with PBS and incubated with Alexa 488 or Alexa 568 conjugated secondary antibodies (Jackson ImmunoResearch, 1:500 dilution). After washing in PBS, sections were stained using the DNA dye bisbenzimide (Hoechst), washed in PBS and mounted on cover glasses using fluoromount-G.

### Immunoblotting

Immunoblotting analyses of whole cell extracts were performed as described [24]. Briefly, cells were disrupted in lysis buffer containing 1% Triton X-100, and the protein concentration of cleared lysate was performed using the BioRad Protein Assay Dye Reagent. Proteins were separated on using SDS-PAGE, transferred to nitrocellulose membrane (Protran, Schleicher & Schuell), and immunoblotted using antibodies against GFP (NeuroMab) and ERK1/2 (Cell Signaling).

### Statistics

**Figure 1:**
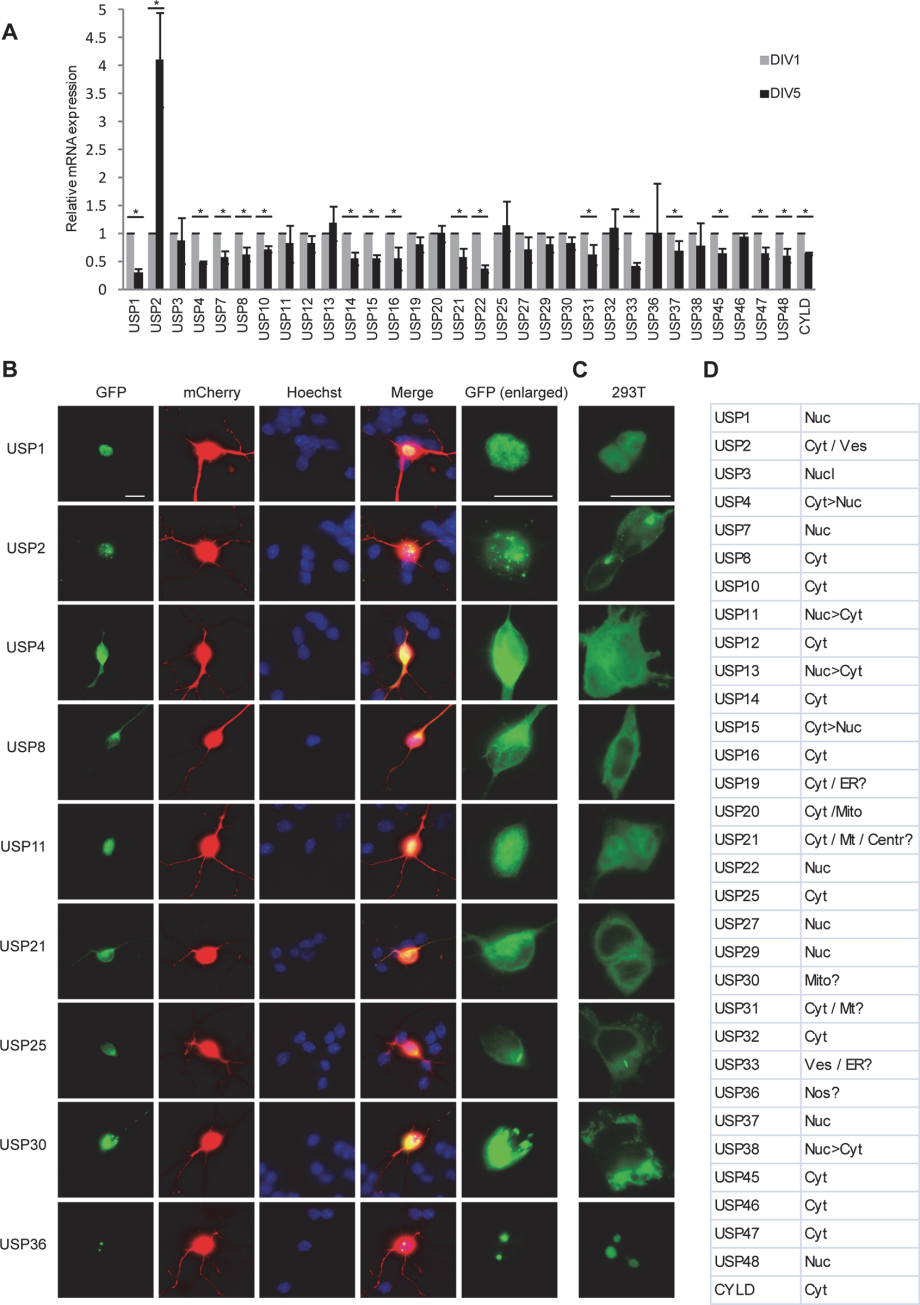
Expression and subcellular locale of USPs in granule neurons. **A**. Analysis of USP gene expression in primary granule neurons. Neurons were isolated from P6 rat pups. After one or five days in vitro (DIV), total neuronal mRNA was isolated, reverse transcribed to cDNA, and analyzed by quantitative RT-PCR using *gapdh* as a control gene. Asterisks indicate significant changes in expression between DIV1 and DIV5 (P<0.05, t-test). **B**. Subcellular localization of neuronally expressed USPs in primary granule neurons. Cells isolated from P6 rat pups were cultured in vitro and at DIV2 transfected with expression plasmids encoding the indicated GFP-tagged USP. To visualize the entirety of the neuron, cells were cotransfected with plasmids encoding mCherry. Two days after transfection, cells were fixed and subjected to immunocytochemical analyses using the GFP and dsRED antibodies. Staining with the DNA dye bisbenzimide (Hoechst) was used to visualize the cell nucleus. An enlarged view of the localization of each USP in neurons is shown in the indicated panel. Bar = 10μm. **C**. Subcellular localization of neuronally expressed USPs in 293T cells. **D**. Summary of the subcellular localization of USPs in neurons. Abbreviations: Cent—Centrosome; Cyt—cytoplasm; ER—endoplasmic reticulum; Mito—mitochondria; Mt—microtubules; Nuc—nucleus; Nos—nucleolus; Ves—vesicles.

**Figure 2:**
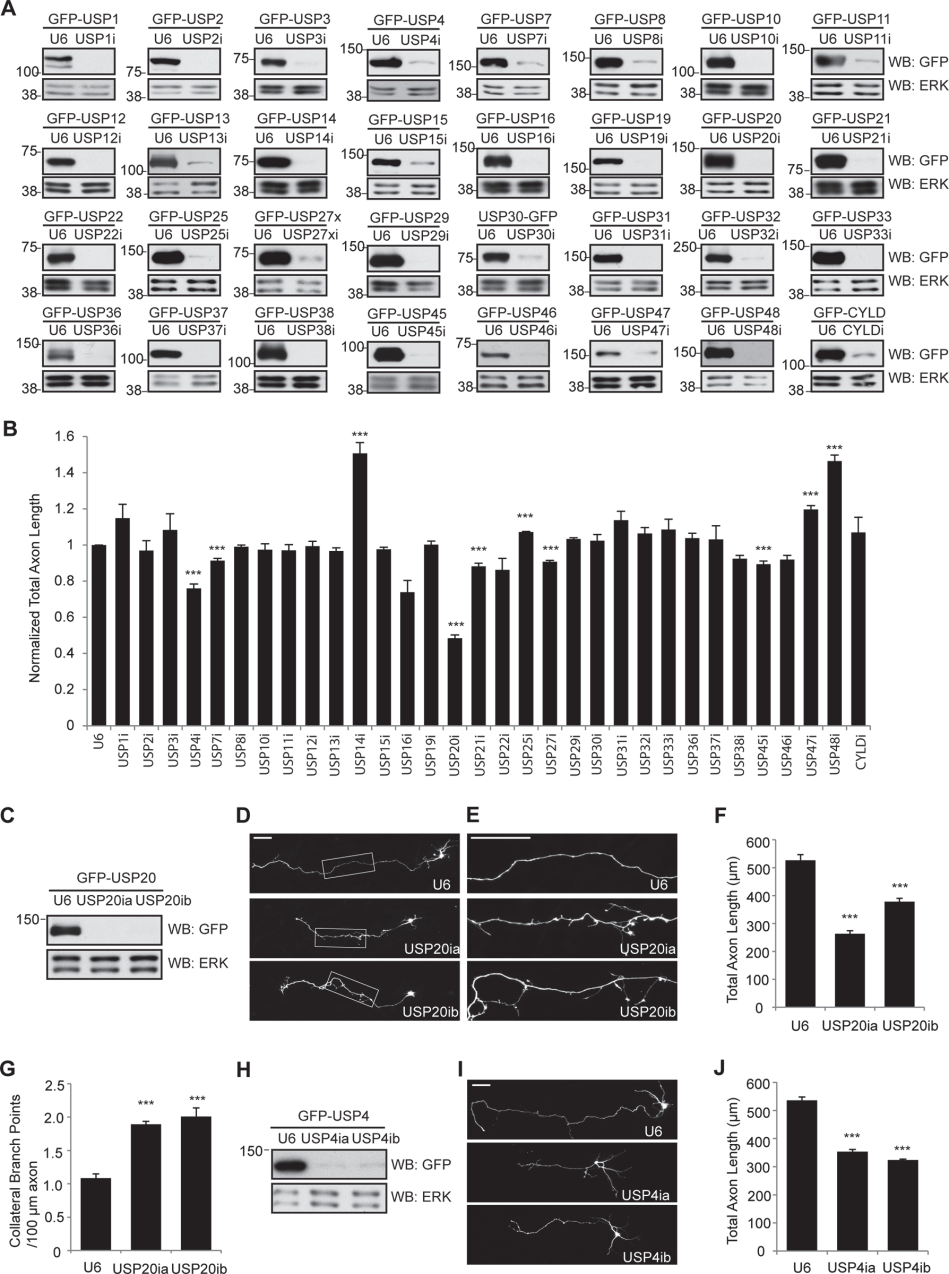
USP RNAi screen reveals functions for USP4 and USP20 in granule neuron axon development. **A**. Generation of a plasmid-based shRNA library targeting neuronally expressed USPs. Target sequences of 32 candidate USPs were inserted into a pBS/U6 backbone vector, and the efficiency of the shRNA constructs was analyzed by cotransfecting 293T cells with the indicated GFP-USP expression constructs together with empty vector (U6) or shRNA-encoding plasmids. Whole cell lysates were analyzed by immunoblotting using the GFP and ERK antibodies, the latter to serve as loading control. **B**. Effect of knockdown of 32 USP genes on axon growth. Cerebellar granule neurons prepared from P6 rat pups were transfected with a GFP-expression plasmid and the indicated RNAi plasmid. Four days later, neurons were fixed and subjected to immunocytochemistry using the GFP antibody, and total axon length in transfected neurons was determined. Total axon length in granule neurons transfected with each RNAi plasmid was normalized to the total length in control U6-transfected granule neurons. A total of 3665 cells were counted. Asterisks indicate statistically significant effects (P<0.005, t-test) **C**. Lysates of 293T cells transfected with the GFP-USP20 expression plasmid together with the indicated USP20 RNAi or control U6 RNAi plasmid were immunoblotted with the indicated antibodies. **D-G**. USP20 knockdown significantly decreased the total axon length (*P*<0.001, ANOVA, n = 3, 279 neurons) and led to aberrant axon branching (*P*<0.001, ANOVA, n = 3, 212 neurons) in granule neurons. Granule neurons were transfected with the USP20 RNAi plasmids or the U6 control plasmid as in panel 2B. Panel E shows an enlarged view of the boxed area in D. **H**. Lysates of 293T cells transfected with GFP-USP4 together with the U6 or indicated USP4 RNAi plasmid were immunoblotted with the indicated antibodies. **I-J**. USP4 RNAi significantly decreased total axon length in granule neurons (*P*<0.001, ANOVA, n = 3, 234 neurons counted). Granule neurons were transfected with the USP4 RNAi plasmids or the U6 control plasmid as in Fig. 2B. The size of all scale bars is 50μm.

**Figure 3:**
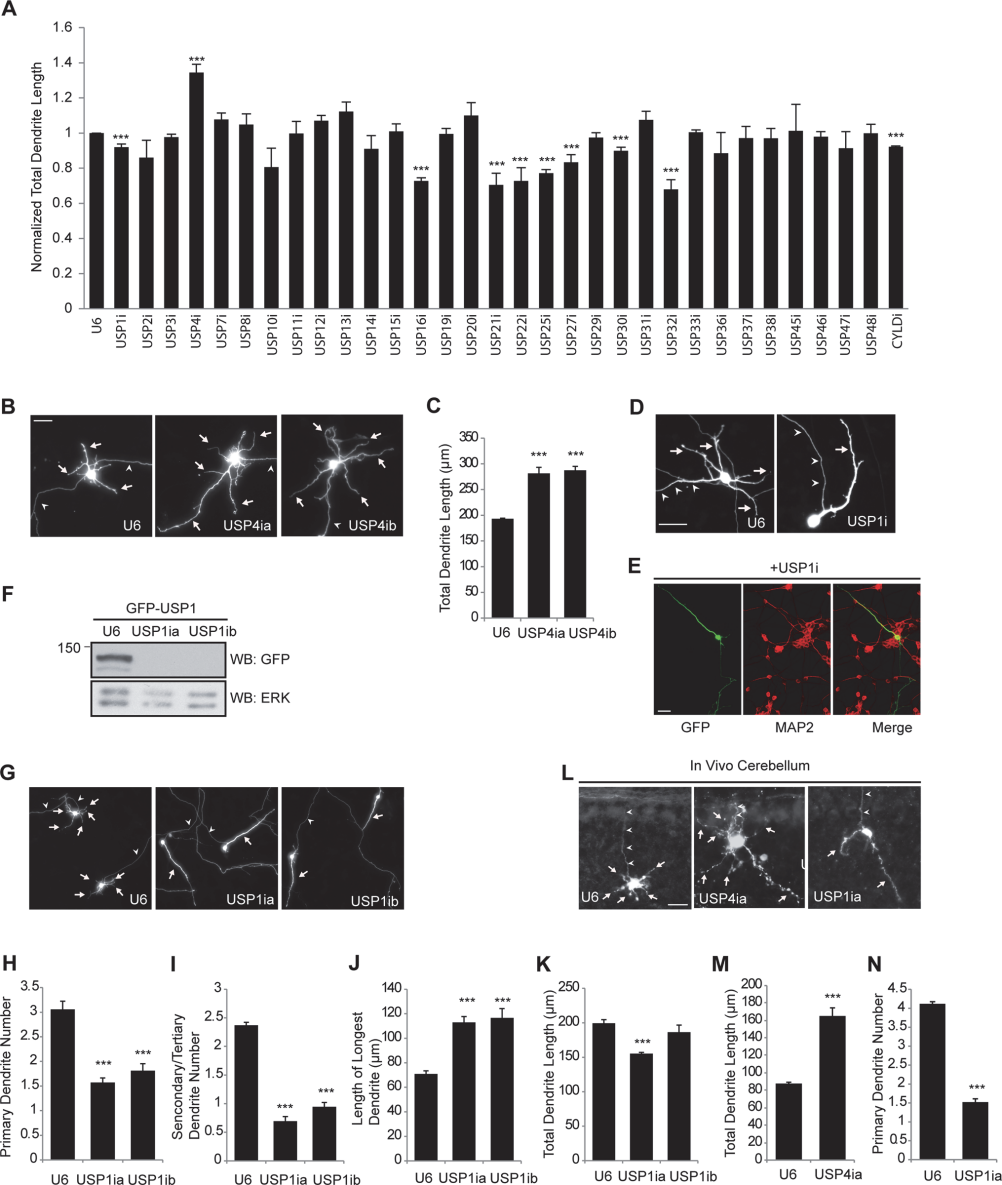
Regulation of granule neuron dendrite morphogenesis by USP1 and USP4. **A**. Cerebellar granule neurons were prepared as in Fig. 2B, transfected at DIV2 and analyzed at DIV5. Total dendrite length in granule neurons transfected with each RNAi plasmid was normalized to the total dendrite length of control U6 transfected neurons. Asterisks indicate statistically significant effects (P<0.005, t-test). **B, C**. USP4 knockdown stimulates dendrite growth. Granule neurons were transfected as in panel A, and analyzed for dendrite length. Arrows and arrowheads indicate dendrites and axons, respectively. USP4 knockdown significantly increased total dendrite length (*P*<0.001, ANOVA, n = 3; 209 cells counted). **D-E**. USP1 knockdown simplifies granule neuron dendrite arbors. Neurons transfected with GFP and USP1 RNAi or U6 control plasmid were subjected to immunocytochemistry using the GFP and the MAP2 antibodies. Arrows and arrowheads indicate dendrites and axons, respectively. **F**. Lysates of 293T cells transfected with GFP-USP1 together with U6 or the indicated USP1 RNAi plasmid were immunoblotted with the indicated antibodies. **G-K**. Granule neurons were transfected as in A with the USP1 RNAi plasmid or control U6 plasmid. USP1 knockdown significantly reduced primary and secondary/tertiary dendrite numbers and increased length of longest dendrite (*P*<0.001 for 3H; *P*<0.001 for 3I; *P*<0.005 for 3J, ANOVA, n = 3; 206 cells), but had little or no effect on total dendrite length. Arrows and arrowheads indicate dendrites and axons, respectively. **L-N**. USP1 and USP4 RNAi regulate dendrite development *in vivo*. P4 rat pups were injected with plasmids encoding GFP together with the control U6 RNAi plasmid or the indicated RNAi plasmid targeting USP1 or USP4. Dendrite length and primary dendrite number of transfected cells were determined following immunohistochemistry of coronal sections of GFP-positive cerebella. For panel 3M, 81 cells and for panel 3N, 131 cells were counted. USP4 knockdown significantly increased total granule neuron dendrite length (*P*<0.005, *t*-test) and reduced primary granule neuron dendrite number (*P*<0.001, *t*-test) *in vivo*. The size of all scale bars is 20μm. Arrows and arrowheads indicate dendrites and axons, respectively.

Student's t-test was used in experiments comparing two groups. Pairwise comparison within multiple groups was done by ANOVA followed by the Bonferroni *post hoc* test, except in Figs. 1A, 2B, and 3A, where the unpaired t-test was used. All data in histograms are presented as mean ± SEM and obtained from three independent experiments, except USP45i in Fig. 3A (n = 2).

## Results and Discussion

To characterize the roles of USP enzymes in the brain, we first assessed the expression of all USP family members in granule neurons of the rodent cerebellar cortex in quantitative RT-PCR analyses. Of 50 assayed USP genes, we selected 32 candidates for further analyses, and interrogated their expression in granule neurons upon maturation. We measured mRNA levels of the selected USP genes in neurons prepared from postnatal day 6 (P6) rat pups and cultured for one day in vitro (DIV1), when neurons are polarizing and actively extending neuronal processes [25]. We also monitored USP mRNA levels in granule neurons from P6 rat pups and cultured for five days in vitro (DIV5), when granule neurons have established axons and dendrite arbors [23]. The mRNA levels of most of the 32 selected USP genes remained constant or decreased slightly with maturation, suggesting that the expression of USP genes is higher in neurons undergoing morphological changes (Fig. 1A). Notably, however, the mRNA levels of USP2 increased substantially in granule neurons with maturation (Fig. 1A). These results define a set of 32 USP family members that are expressed in granule neurons during the time that they undergo maturation.

We next characterized the subcellular localization of the neuronally expressed USPs. To facilitate surveying the subcellular distribution of the set of USPs, we generated a library of USP-GFP fusion expression plasmids. We transfected primary granule neurons with the USP-GFP expression plasmids together with an expression plasmid encoding mCherry, the latter to visualize the transfected granule neurons. In immunocytochemical analyses, the majority of USPs were expressed in multiple compartments in granule neurons including the nucleus and cytoplasm (Figs. 1B and S1, S2.). However, the localization of a number of USPs was restricted to specific subcellular locales (Figs. 1B, 1D, and S1, S2). Intriguingly, USP25 was localized to elongated structures reminiscent of cilia, but these structures failed to label with the cilia marker adenylyl cyclase 3 (data not shown). To explore whether the observed localization patterns were specific to neurons, we also characterized the localization of the GFP-USPs in 293T cells (Figs. 1C and S1, S2). These analyses suggested that there were few differences in the localization of USPs in 293T cells and primary neurons. A notable exception was USP20, which in addition to cytoplasmic localization was consistently localized at mitochondria in neurons but not in 293T cells (Figs. 1D and S3), suggesting that the localization of USP20 may be regulated in a cell-type-specific manner. Together, these results highlight the diverse spatial distribution of USPs in neurons.

The robust expression of the 32 USP family members in granule neurons led us next to determine their biological functions in granule neurons in the rodent cerebellar cortex. To characterize the roles of the neuronally expressed USPs, we employed a plasmid-based RNAi-induced knockdown strategy [21], [26]. We generated a library of RNAi plasmids expressing short hairpin RNAs (shRNAs) targeting the 32 neuronally expressed USPs. We first confirmed that expression of the shRNAs induced the knockdown of the targeted USPs (Fig. 2A). We next characterized the effect of knockdown of the 32 USPs on the morphogenesis of granule neurons. Granule neurons were prepared from postnatal day 6 (P6) rat pups and transfected with the control U6 RNAi plasmid or RNAi plasmid encoding shRNAs targeting each of the 32 USPs. Four days later, we subjected granule neurons to immunocytochemical and morphometric analyses to measure the effect of USP knockdown on the morphology of axons.

Knockdown of most of the 32 USPs had little or no effect on axon growth (Fig. 2B). Depletion of USP14 and USP48 increased the length of axons in granule neurons (Fig. 2B), suggesting that USP14 and USP48 restrict the growth of axons. In contrast to the axon growth-promoting effect of USP14 knockdown and USP48 knockdown, depletion of USP4 or USP20 substantially reduced the growth of granule neuron axons (Figs. 2B-2J). The impairment of axon growth upon knockdown of USP4 and USP20 was observed upon expression of shRNAs targeting distinct regions of USP4 and USP20, respectively (Figs. 2C-2J). Notably, USP20 knockdown increased the number of axon branches concomitantly with the reduction in total axon length (Figs. 2E-2G). By contrast, knockdown of USP4 had little or no effect on axon branching (Fig. 2I). These results suggest that although USP4 and USP20 share the capacity to promote the growth of axons, they have differential effects on other aspects of axon morphogenesis.

Having analyzed the roles of USPs in axon development, we next characterized the functions of the neuronally expressed USPs in dendrite morphogenesis. To rule out possible effects on dendrite arbor formation as a result of alterations in neuronal polarization, we induced the knockdown of the 32 USPs in granule neurons two days after plating at a time when neurons are already polarized [25], and characterized the morphogenesis of dendrites in these neurons three days later. Just as in the case of axon development, knockdown of most USPs had little or no effect on the growth of granule neuron dendrites (Fig. 3A). Depletion of a few USPs reduced the growth of dendrites (Fig. 3A). In particular, knockdown of USP21, a microtubule-associated deubiquitinating enzyme (DUB) that mediates nerve growth factor (NGF)-induced neurite outgrowth in PC12 cells [27], led to reduced total dendrite length (Fig. 3A). Although depletion of USP22, USP25 and USP27x reduced the length of dendrites in granule neurons (Fig. 3A), cell survival was also impaired in these neurons (data not shown), suggesting that the effect of knockdown of these USPs on dendrite growth may be secondary to reduced cell survival.

Remarkably, knockdown of USP4 substantially increased the length of dendrites in granule neurons (Fig. 3A), an effect that was mimicked by two distinct shRNAs targeting USP4 (Figs. 3B and 3C). Thus, our results suggest that USP4 has opposing effects on the morphogenesis of axons and dendrites. USP4 promotes the growth of axons and concomitantly restricts the growth of dendrites.

Depletion of USP1 had little or no effect on axon development but led to striking effects on the morphogenesis of dendrites. Dendrite arbors were simplified in USP1 knockdown granule neurons, induced by two distinct shRNAs, leading to reduced number of primary dendrites and secondary branches (Figs. 3D-3I). Remarkably, the length of the primary dendrite in USP1 knockdown neurons was significantly increased (Figs. 3G and 3J). Consequently, USP1 knockdown had little or no effect on the total length of dendrites in granule neurons (Fig. 3K). These results suggest that USP1 regulates the architecture of granule neuron dendrite arbors.

Having identified functions for USP1 and USP4 in dendrite development in primary granule neurons, we next assessed whether these USPs are required for proper granule neuron dendrite development in the cerebellum *in vivo*. We employed an *in vivo* RNAi approach o induce the knockdown of USP1 and USP4 in P4 rat pups and analyzed animals five days later at P9 [12], [28]. Sections of the cerebellum were subjected to immunohistochemical analyses. Granule neuron somas in the internal granule layer (IGL) in control animals displayed the characteristic T-shaped parallel fiber axons in the molecular layer and well-defined dendrite arbors in the IGL (Fig. 3L). We found that granule neuron dendrite arbors were substantially more elaborate in USP4 knockdown animals, leading to significantly increased total dendrite length (Fig. 3L and 3M). By contrast, granule neuron dendrite arbors were profoundly simplified in USP1 knockdown animals, leading to significant reduction in the number of primary dendrites in these animals (Fig. 3L and 3N). Together, our findings highlight a critical role for USP1 and USP4 in sculpting granule neuron dendrite arbors in the mammalian cerebellum.

**Figure 4:**
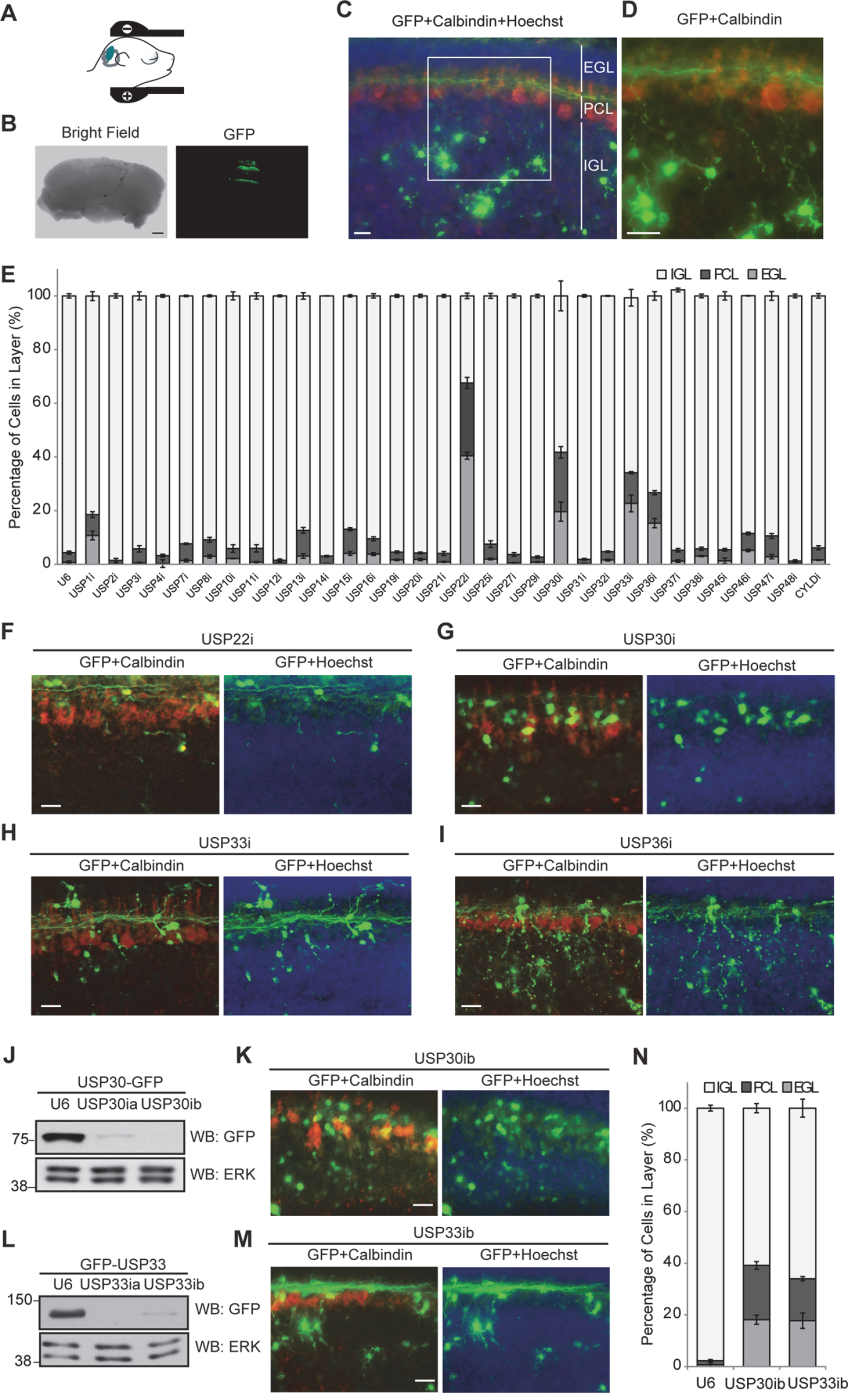
USP30 and USP33 are required for granule neuron migration in the rodent cerebellar cortex *in vivo*. **A-D**. Cerebella of P4 rat pups were injected with expression plasmids encoding synapsin-mCitrin and the U6 control and subjected to electroporation. Pups were sacrificed five days later, at P9, and coronal sections of cerebella were analyzed by immunohistochemistry. Transfected neurons and Purkinje cells were visualized with the GFP (green) and calbindin antibodies (red), respectively. Staining with the DNA dye bisbenzimide (Hoechst) was used to label cell nuclei. Panel D shows an enlarged view of the boxed area in panel C. Bar in panel B = 500μm. **E**. An *in vivo* RNAi screen targeting USPs in the intact mammalian brain. P4 rat cerebella were subjected to *in vivo* electroporation as in panels A-D, and neuronal migration was analyzed by counting the number of neurons residing in the indicated layers. For each RNAi condition, at least three different brains were used for quantification. In total, 21536 cells were counted. **F-I**. Representative images showing impaired migration of neurons in rat pups transfected with RNAi targeting USP22, USP30, USP33 or USP36. **J**. Lysates of 293T cells transfected with USP30-GFP together with U6 or the indicated USP30 RNAi plasmid were immunoblotted with the indicated antibodies. **K, N**. USP30 knockdown impairs neuronal migration in the cerebellum. Cerebella of P4 rat pups were subjected to *in vivo* electroporation as in panels A-D. **L**. Lysates of 293T cells transfected with GFP-USP33 together with U6 or the indicated USP33 RNAi plasmid were immunoblotted with the indicated antibodies. **M, N**. USP33 knockdown impairs neuronal migration in the cerebellum. Cerebella of P4 rat pups were subjected to in vivo electroporation as in panels A-D. EGL—external granule layer; PCL—Purkinje cell layer; IGL—internal granule layer. The size of all scale bars is 20μm, unless otherwise noted.

Besides axon and dendrite morphogenesis, the migration of neurons is a prerequisite for proper neuronal connectivity in the mammalian brain. Ubiquitin ligases have been implicated in the control of granule neuron migration in the cerebellum [10]. However, the role of deubiquitinases in granule neuron migration in the mammalian cerebellum has remained unexplored. We performed an *in vivo* RNAi screen of the 32 neuronally expressed USPs in the rodent cerebellum to determine the functions of the USPs in the migration of granule neurons from the external granule layer (EGL) to the internal granule layer (IGL). In these experiments, we employed the *in vivo* electroporation approach to introduce each of the 32 USP RNAi plasmids together with an expression plasmid encoding mCitrin under the control of the synapsin I promoter, the latter to label transfected neurons specifically in these animals (Fig. 4A and 4B). At this stage, sequential waves of precursor cell differentiation in the EGL generate granule neurons, which subsequently migrate inward through the molecular layer and past Purkinje cells to their final destination in the IGL [1], [3]. Pups were returned to their mothers and sacrificed five days later at P9. Coronal sections of the cerebellum were subjected to immunohistochemical analyses using antibodies to Calbindin and GFP to label Purkinje neurons and the transfected granule neurons (Fig. 4C and 4D). In our analyses of granule neuron migration, the position of granule neurons was designated in the EGL, IGL, or Purkinje cell layer based on the location of granule neurons relative to the soma of Purkinje cells (Fig. 4C and 4D).

In rat pups transfected with the control U6 plasmid, the majority of GFP-positive granule neurons had migrated into the IGL at P9 and displayed their associated characteristic T-shaped parallel fiber axons in the molecular layer (Fig. 4C and 4D). Just as in the case of axon and dendrite morphogenesis in granule neurons, knockdown of most members of the 32 neuronally expressed USPs had little or no effect on the positioning of granule neurons in the cerebellum *in vivo* (Figs. 4E and S4, S5). However, the knockdown of four USPs, USP22, USP30, USP33, and USP36, robustly impaired granule neuron migration with large numbers of granule neurons in knockdown animals positioned in the EGL or Purkinje cell layer rather than the IGL (Fig. 4E-4I). Because USP22 knockdown had an effect on cell survival in primary granule neurons (data not shown) and USP36 regulates nucleolar and ribosome biogenesis [29] and its knockdown might thus generally affect the health of neurons, we focused further analyses on USP30 and USP33. We generated additional RNAi plasmids encoding shRNAs targeting distinct regions of USP30 and USP33. We found that knockdown of USP30 and USP33 with these shRNAs also robustly impaired the positioning of granule neurons in the cerebellar cortex *in vivo* (Fig. 4J-4N). Together, these findings highlight a critical role for USP30 and USP33 in the migration of granule neurons in the mammalian cerebellum.

In this study, we have defined the roles of 32 ubiquitin-specific proteases (USPs) in cerebellar granule neurons and in the intact mammalian brain. Characterization of a library of expression plasmids encoding GFP fused to each of the 32 USPs has revealed that different USPs localize to distinct subcellular compartments in granule neurons. In RNAi screens of the 32 neuronally expressed USPs in primary granule neurons and the rodent cerebellum *in vivo*, we have discovered novel functions for distinct USPs in granule neuron morphogenesis and positioning. USP14 and USP48 restrict the growth of axons, whereas USP20 promotes the growth of axons and inhibits their branching in granule neurons. USP4 promotes the growth of granule neuron axons and concomitantly restricts the growth and elaboration of dendrite arbors. USP1 controls the architecture of granule neuron dendrites. Finally, USP30 and USP33 promote the migration of granule neurons in the rodent cerebellum *in vivo*. Our findings reveal novel functions for distinct USPs in neuronal morphogenesis and positioning in the mammalian cerebellum.

The identification of novel functions for USPs in granule neuron morphogenesis and positioning advances our understanding of the mechanisms that establish neuronal connectivity in the mammalian brain. Our studies highlight the key role of distinct USP members in different aspects of neuronal development. How might the specificity of USP function arise? In the case of ubiquitin ligases, their differential localization plays a critical role in determining their specificity of function [9]. For example, the ubiquitin ligase Cdh1-APC operates in the nucleus to limit granule neuron axon growth [30], [31], whereas the related ubiquitin ligase Cdc20-APC acts at the centrosome to promote the growth and elaboration of granule neuron dendrite arbors [11], [32]. On the other hand, the ubiquitin ligase Cul7^Fbxw8^ localizes at the Golgi apparatus and thereby drives the growth of granule neuron dendrites [13]. These studies suggest the possibility that spatial control of deubiquitinating enzymes may also specify their functions in brain development. Thus, the characterization of the subcellular localization of USPs may provide critical clues for the specificity of USP function in neuronal morphogenesis and connectivity.

Among the USPs that we have implicated in the regulation of granule neuron development, USP20 promotes the growth of axons and simultaneously restricts the formation of axon branches. Thus, USP20 may contribute to the morphology of granule neuron axons, which form long parallel fibers that are relatively devoid of branches. Although USP20 reportedly localizes to intracellular vesicles of the endosomal shuttling pathway in cell lines [33], we have found that USP20 localizes at mitochondria in granule neurons and in particular within granule neuron axons (S3 Fig.). Interestingly, the protein kinases LKB1 and NUAK1 promote axon branching and presynaptic development of cortical neurons via the immobilization of axonal mitochondria [34]. It will be therefore interesting in the future to explore whether USP20 and the LKB1/NUAK1 pathway intersect in the regulation of axon branching.

Whereas USP20 selectively promotes the growth of axons with little or no effect on dendrite growth, USP4 promotes the growth of granule neuron axons and concomitantly suppresses the growth of their dendrites. Thus, USP4 may contribute to the unique morphological appearance of neurons with long axons and short dendrites. The role of USP4 in the coordinate regulation of granule neuron axon and dendrite growth is reminiscent of the function of the FOXO transcription factors, and in particular the brain-enriched protein FOXO6, in the morphogenesis of granule neurons [25]. The broad localization of USP4 in both the cytoplasm and nucleus is consistent with the possibility that the functions of USP4 and the FOXO proteins may be coordinated in the regulation of granule neuron morphogenesis.

Our study suggests that USP30 and USP33 may play critical roles in granule neuron migration in the rodent cerebellum *in vivo*. Intriguingly, USP30 appears to be specifically localized to the mitochondria in granule neurons. USP30 has been recently implicated in the control of mitophagy [35]. These observations raise the possibility that the regulation of mitochondrial morphogenesis and function may contribute to neuronal migration in the mammalian brain. USP33 appears to be localized in vesicular structures in the cytoplasm in granule neurons. Interestingly, USP33 regulates centrosome biogenesis in proliferating cells [36]. In view of the key role of the centrosome in neuronal migration [37]—[38], it will be important to determine whether USP33 regulates granule neuron migration via a mechanism that operates at the centrosome.

USPs often directly interact with and counteract the activity of ubiquitin ligases in the regulation of biological processes [20]. Therefore, the interplay of USP function with ubiquitin ligases in the regulation of neuronal morphogenesis and connectivity represents another area of future research. It will be interesting to determine whether and how the function of USP4, USP14, USP20 and USP48 is coordinated with Cdh1-APC in the control of granule neuron axon morphogenesis. Likewise, whether USP4 and USP1 influence the ability of Cdc20-APC and Cul7^Fbxw8^ to drive the elaboration of granule neuron dendrite arbors will be interesting to explore. Finally, it will be interesting to assess whether USP30 and USP33 function intersects with the activity of the ubiquitin ligase Siah in the regulation of granule neuron migration in the cerebellum [10].

In summary, our study provides a comprehensive survey of the expression and function of USPs in granule neurons of the rodent cerebellar cortex. Among the 32 neuronally expressed USPs, we have identified novel functions for several USPs including USP1, USP4, USP20, USP30, and USP33 in the regulation of granule neuron morphogenesis and positioning in the mammalian cerebellum.

## Conclusions

A comprehensive analysis of ubiquitin-specific proteases (USPs) reveals that specific USPs with distinct spatial localizations including USP1, USP4, USP20, USP30 and USP33 harbor key functions in the control of neuronal morphogenesis and positioning in the mammalian cerebellum.

## Supporting Information

S1 Fig Subcellular localization of USPs in primary granule neurons and 293T cells. Cells were transfected and analyzed as in Fig. 1B. Bar = 10μm
(TIF)

S2 Fig Subcellular localization of USPs in primary granule neurons and 293T cells. Cells were transfected and analyzed as in Fig. 1B. Bar = 10μm
(TIF)

S3 Fig USP20 localizes to mitochondria in neurons. Granule neurons were transfected with plasmids encoding GFP-USP20 and mCherry (A) or Mito-dsRed (B). Cells were analyzed using immunocytochemistry as in Fig. 1B. Bar = 10μm.
(TIF)

S4 Fig RNAi screen of USPs in neuronal migration in the rodent cerebellar cortex. Representative images of immunohistochemical analyses of coronal sections of cerebella subjected to *in vivo* electroporation with synapsin-promoter mCitrin, and the indicated USP RNAi. Purkinje cells were labeled with Calbindin (red), and transfected cells with GFP (green). Bar = 20μm.
(TIF)

S5 Fig RNAi screen of USPs in neuronal migration in the rodent cerebellar cortex. Representative images of immunohistochemical analyses of coronal sections of cerebella subjected to *in vivo* electroporation with synapsin-promoter mCitrin, and the indicated USP RNAi. Purkinje cells were labeled with Calbindin (red), and transfected cells with GFP (green). Bar = 20μm.
(TIF)

S1 Table Target sequences for USP shRNA constructs. (PDF)

S2 Table Sequences of RT-PCR primers. (PDF)
