# Correction: Regulation of Neuronal Morphogenesis and Positioning by Ubiquitin-Specific Proteases in the Cerebellum

**DOI:** 10.1371/journal.pone.0133943

**Published:** 2015-07-20

**Authors:** The PLOS ONE Staff

Due to a typesetting error, the Funding section is missing in this article. The Funding section should read as follows: "The work was supported by NIH grant NS051255 (A.B.) and grants from the Swedish Cultural Foundation in Finland (J.A.) and Magnus Ehrnrooth Foundation (J.A.)." The publisher apologizes for this error.
