# Correction: Correction: Regulation of Neuronal Morphogenesis and Positioning by Ubiquitin-Specific Proteases in the Cerebellum

**DOI:** 10.1371/journal.pone.0135535

**Published:** 2015-08-05

**Authors:** The PLOS ONE Staff

## Notice of Republication

This article was republished on July 27, 2015, to change the byline of the correction, which should have been The *PLOS ONE* Staff, and to note the publisher as responsible for the error. The publisher apologizes for the errors. Please download this article again to view the correct version. The originally published, uncorrected article and the republished, corrected article are provided here for reference.

## Supporting Information

S1 File Originally published, uncorrected article. (PDF)

S2 File Republished, corrected article. (PDF)

## References

[1] The *PLOS ONE* Staff (2015) Correction: Regulation of Neuronal Morphogenesis and Positioning by Ubiquitin-Specific Proteases in the Cerebellum. PLoS ONE 10(7): e0133943 doi:10.1371/journal.pone.0133943 26191798 10.1371/journal.pone.0133943 PMC4508107

