# Supplementary figures and images for: Regulation of Neuronal Morphogenesis and Positioning by Ubiquitin-Specific Proteases in the Cerebellum
Source: PLoS One. 2015 Jan 21;10(1):e0117076. doi: 10.1371/journal.pone.0117076 (PMC4301861; doi:10.1371/journal.pone.0117076)

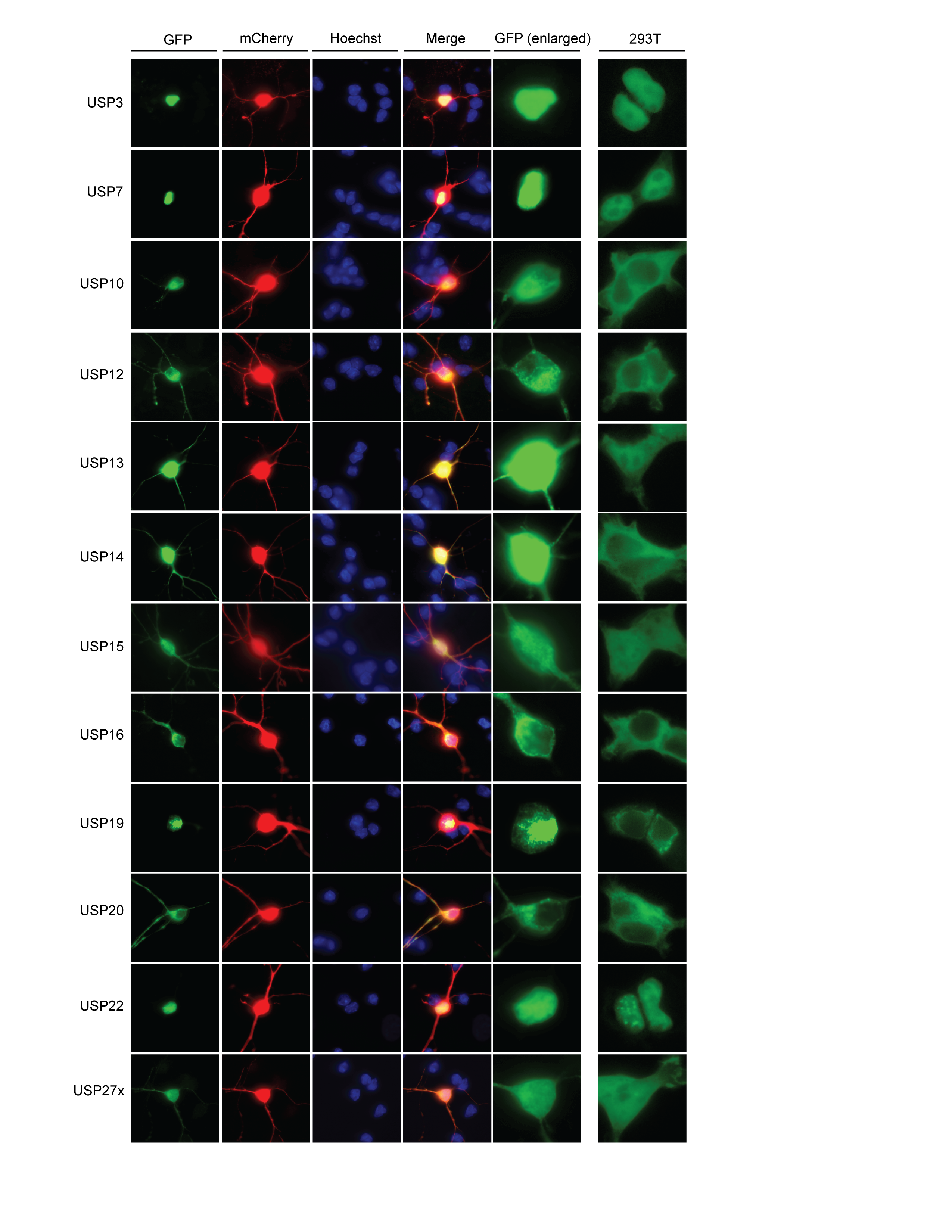

Supplement: S1 Fig — Cells were transfected and analyzed as in Fig. 1B. Bar = 10μm (TIF) [file pone.0117076.s001.tif]

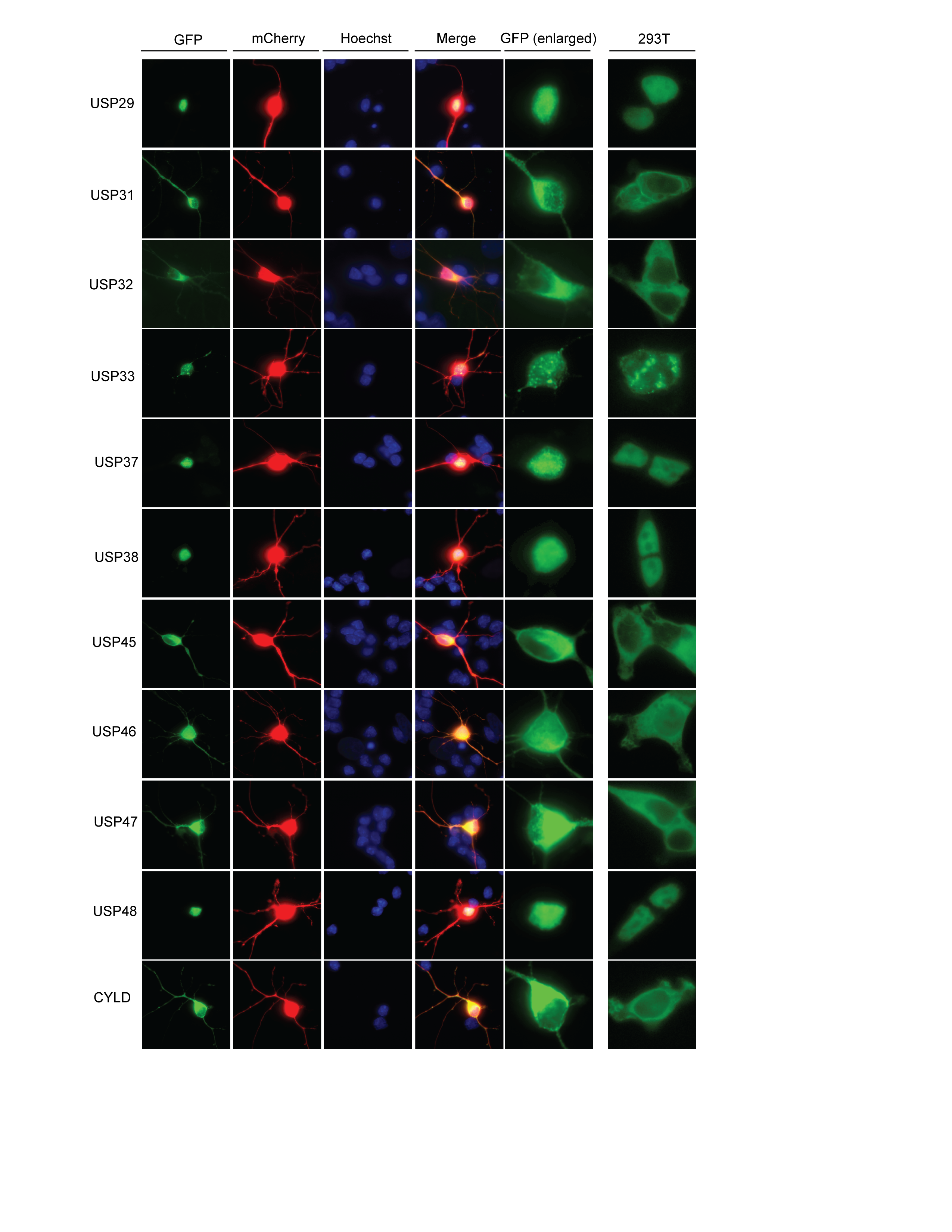

Supplement: S2 Fig — Cells were transfected and analyzed as in Fig. 1B. Bar = 10μm (TIF) [file pone.0117076.s002.tif]

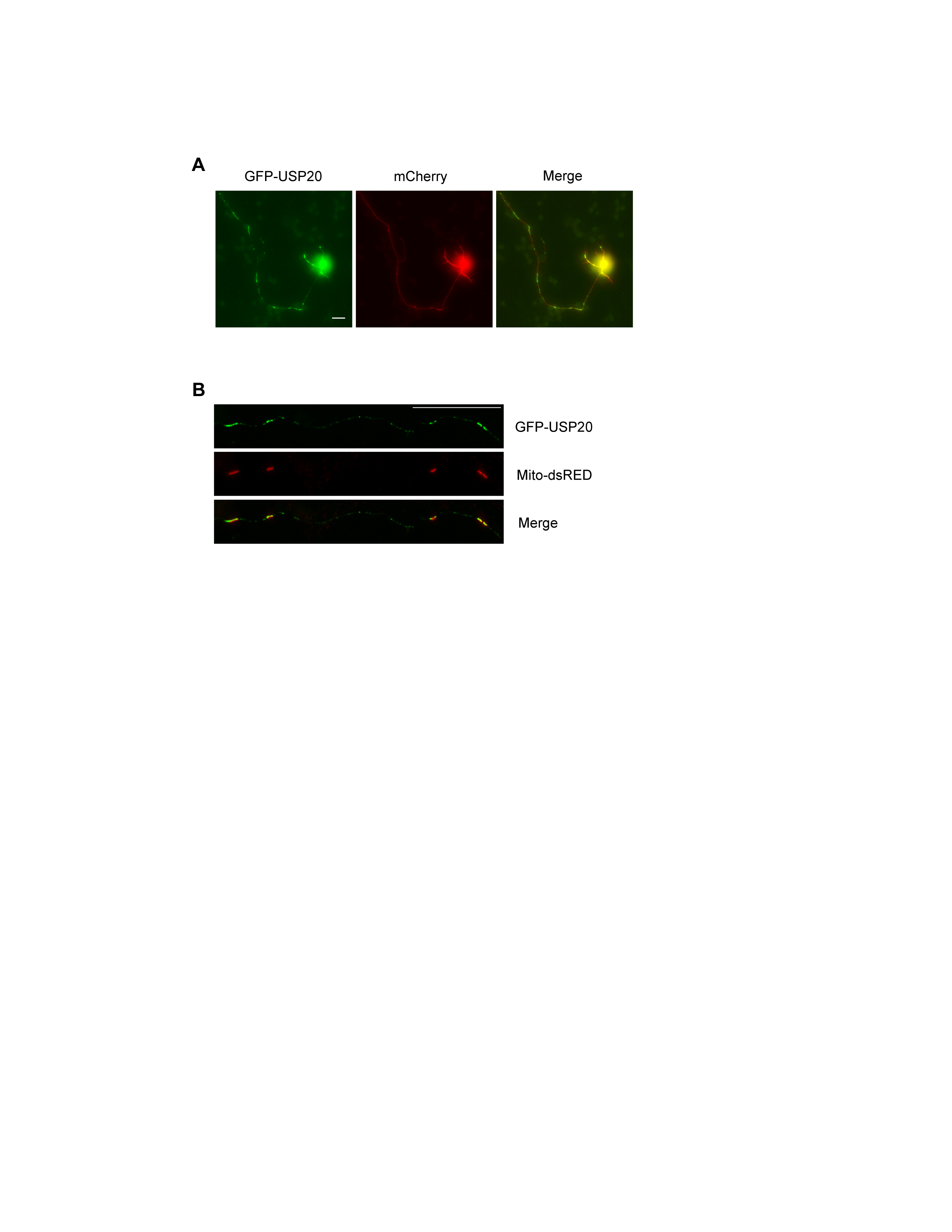

Supplement: S3 Fig — Granule neurons were transfected with plasmids encoding GFP-USP20 and mCherry (A) or Mito-dsRed (B). Cells were analyzed using immunocytochemistry as in Fig. 1B. Bar = 10μm. (TIF) [file pone.0117076.s003.tif]

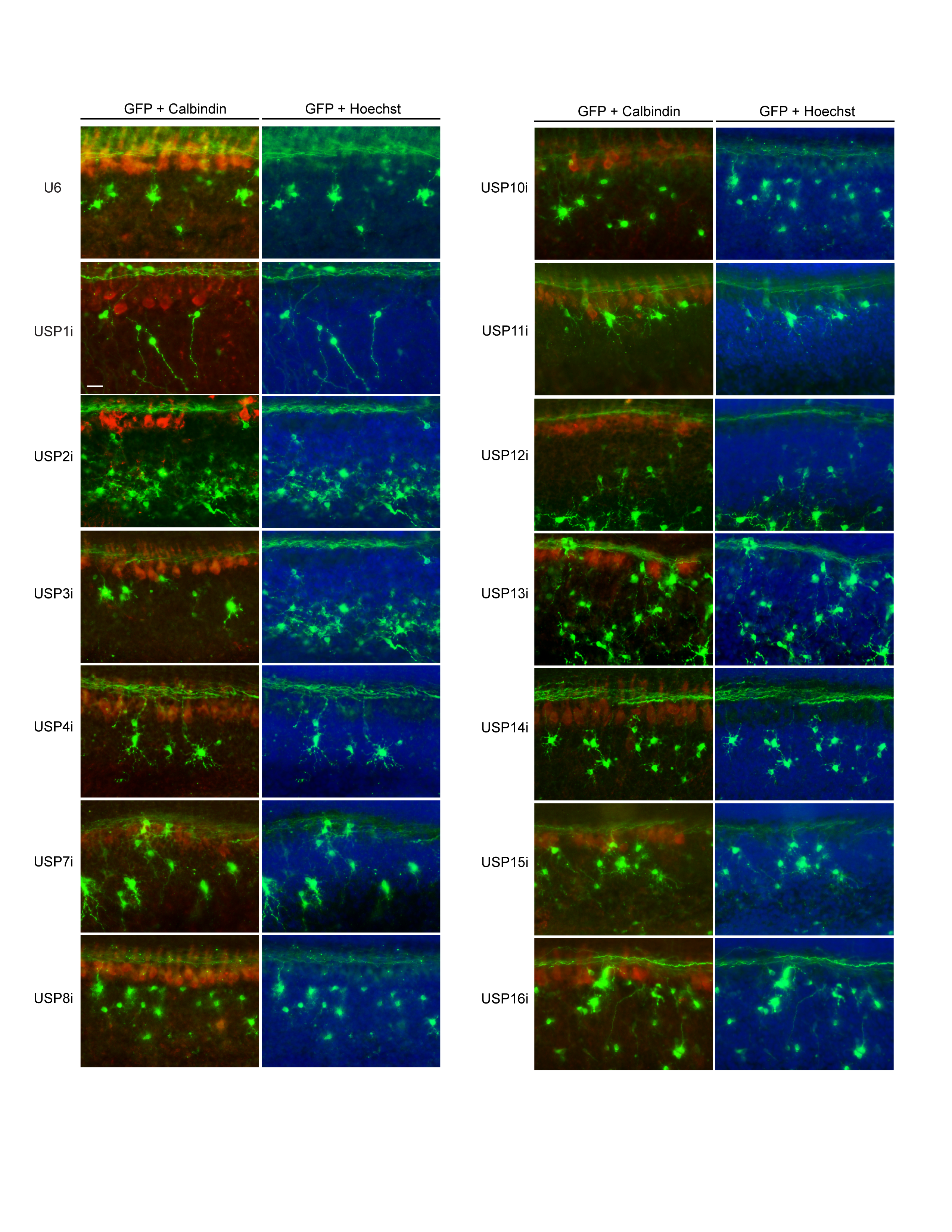

Supplement: S4 Fig — Representative images of immunohistochemical analyses of coronal sections of cerebella subjected to in vivo electroporation with synapsin-promoter mCitrin, and the indicated USP RNAi. Purkinje cells were labeled with Calbindin (red), and transfected cells with GFP (green). Bar = 20μm. (TIF) [file pone.0117076.s004.tif]

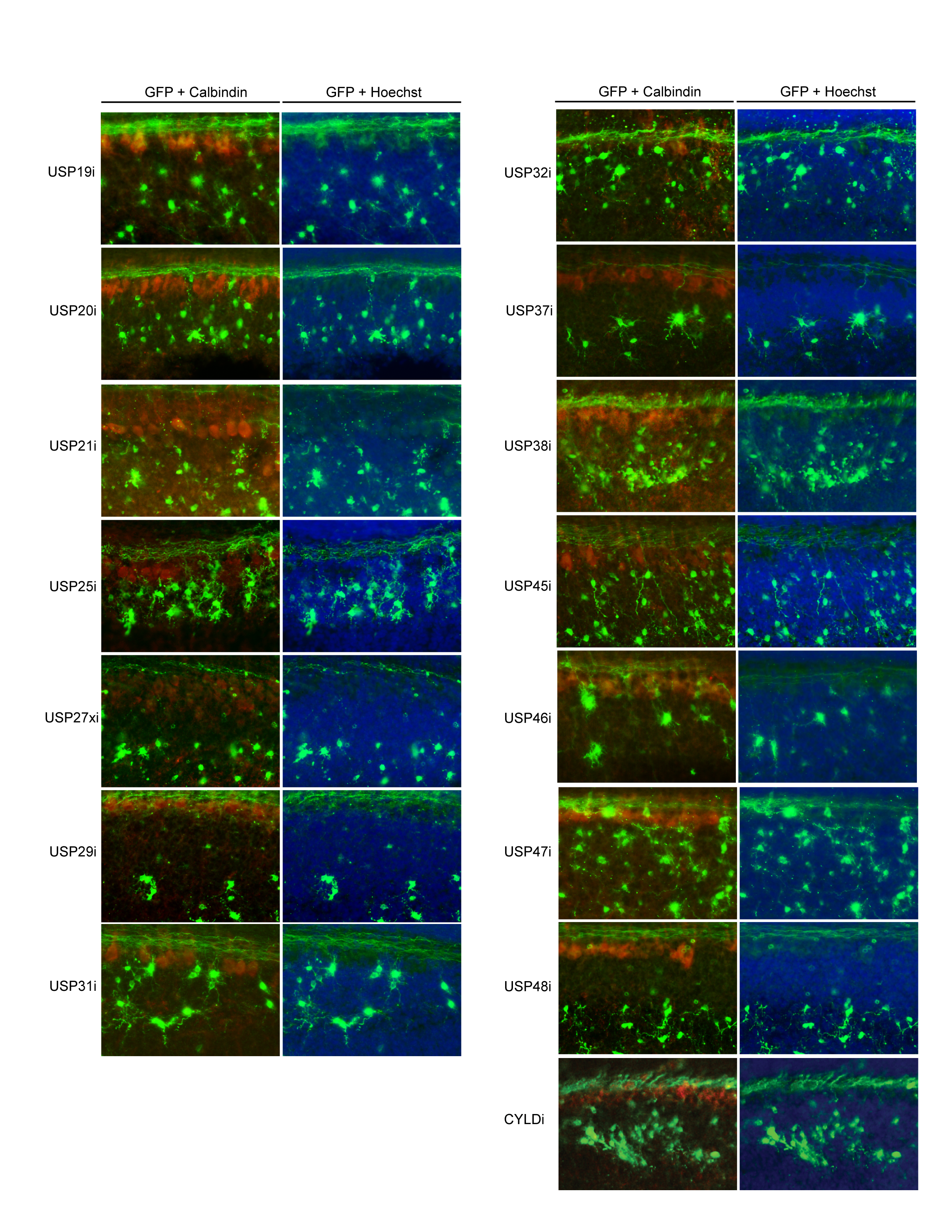

Supplement: S5 Fig — Representative images of immunohistochemical analyses of coronal sections of cerebella subjected to in vivo electroporation with synapsin-promoter mCitrin, and the indicated USP RNAi. Purkinje cells were labeled with Calbindin (red), and transfected cells with GFP (green). Bar = 20μm. (TIF) [file pone.0117076.s005.tif]
