# Supplementary material for: Regulation of Neuronal Morphogenesis and Positioning by Ubiquitin-Specific Proteases in the Cerebellum
Source: PLoS One. 2015 Jan 21;10(1):e0117076. doi: 10.1371/journal.pone.0117076 (PMC4301861; doi:10.1371/journal.pone.0117076)
Supplement: S1 Table — (PDF) [file pone.0117076.s006.pdf]

Construct	Target sequence
USP1ia	GTGCAAAGCTTAAAGGAGTAC
USP1ib	TAACACCCTGAGGGAACTCA
USP2i	GGTGAGCCCATCTGAGTTCAA
USP3i	CAGCAATTCTACAGGAGAATT
USP4ia	CCCAGAATGTGCTAAAGTTTC
USP4ib	CCTGGAATAAATTGCTAAATT
USP7i	CTAGAAATTGTGAGCTACAAA
USP8i	GCATCCACAAGTAAATGTACT
USP10i	CGATGTATCACCTGATGAAGT
USP11i	GATGGACACTATACAACATTT
USP12i	CCCATGAATTCTTAAATTACC
USP13i	TGAGGAAATCGTAGCTATTAT
USP14i	GCACATTGCTTATGTTCTACT
USP15i	ATGTGGTCTGGAAAGTTTAGC
USP16i	AGTGCTTAGAGAACTACTAAA
USP19i	GAAACAGAAGGATCGAGCAAA
USP20ia	TGGCGTGAAGTACTGTAAAGT
USP20ib	GGTGGAGATTGACACTTTCAT
USP21i	GTGCTGTTCTACCAATTGATG
USP22i	GGACAGTCTCAACAATGACA
USP25i	CAGAACCAGGCACCAAAGAAA
USP27xi	GGAGAAGCCTTGAAATTACAA
USP29i	AGCTGAAACTGAACATGGAAA
USP30ia	GTGACAACTGTACAAAGATTG
USP30ib	TGATGGACTTCTACAAGTACC
USP31i	GCATTCACTTAAACAACAATC
USP32i	CTATCAAACATCACAGGAAAG
USP33ia	GGATTCAGTTGGTGAAATAAC
USP33ib	GGCTAGAACAGATAAGAAACC
USP36i	GTGGAGAGAACGCCTATATGT
USP37i	CAGCACTCCATTATCTGTAAA
USP38i	GGACAGACTCCATGAAGAAGA
USP45i	CTTGAGTCTTCGTAAAGTAAA
USP46i	GGAGAATGTGTTGGCATACAA
USP47i	GGATCCAACAAGAAATGCAAA
USP48i	ACTAGAACGAGAAGGTGAAGA
CYLDi	AGTCAATCCTCTGAGAATATA
