# Supplementary material for: Regulation of Neuronal Morphogenesis and Positioning by Ubiquitin-Specific Proteases in the Cerebellum
Source: PLoS One. 2015 Jan 21;10(1):e0117076. doi: 10.1371/journal.pone.0117076 (PMC4301861; doi:10.1371/journal.pone.0117076)
Supplement: S2 Table — (PDF) [file pone.0117076.s007.pdf]

Gene	Primer forward	Primer reverse
USP1	TCAAGTTGTTCCTGCTGCAC	TGCAGCTTCCCTTATCCTTC
USP2	AGATCTTGGTGCTCCACCTG	GTAACCGGACTTCGGCAGTA
USP3	CGCTGTGATGATTTTGTGGT	GGCAGCCGTACTTCCATTTA
USP4	CCTGCAATCAAAATCAAGCA	GGCTCCTGGCAATTATACGA
USP7	CTCCATGCAGTCTTGGTTCA	CTTGCAACACCTCACTCAGC
USP8	AGGCAAGGCTCAGCGAGA	ATCTGGGGCTTGACTTTGTG
USP10	GGCCTCTGAAAAACAGATGG	TGCAGGGTGGCATTAATGTAG
USP11	CAAGAACAAGGTTGGCCATT	TTCCTGAGCCACTTCCAGAT
USP12	TACATGGACCAGCTTCATCG	GATTGGGACCACTTCCACAG
USP13	GGAAGAGCCTGATTTTGCTG	AGGTTATGGTTCGTGGCTTG
USP14	CGCCCTTAAAAGGTATGCAG	CACTCGTTAGCATCCTGCTG
USP15	GGACGTTTTGCACCTCAGTT	TTCAGCAACCACCTTGTCTG
USP16	TCAGGAACAAGCAAGCACAC	TCATTTCCTCTTGGCCATTC
USP19	ACAAAGGGGACACTCCACTG	GCAGTACCAGGCTTCCTCAG
USP20	CCTGTGATCGGGTGTCTACC	CAGGCAGTCTTCCAGTGTGA
USP21	ACACACTGCTTCTGGGCTCT	CATCTGCAAAGGCTTCTGTG
USP22	ACAGACTGCCTGCGAAGATT	CTCTTTGCTGGAGGCCATAA
USP25	GGAAGATGCCTTCCAAATGA	AGGTGGCAATTCAGTGAACC
USP27x	AGCCACATACCAGGCGTTAC	TTCGCTGAGTGTTCAAATCG
USP29	GGATGAGGAGATGGGAGACA	GGGATCTGGATTCTCGAACA
USP30	CCTGTTCGGTTTGACACCTT	TGTGGCAGCTTTCCTAGCTT
USP31	CAGCAAGGGAAAAGATTTGG	GCCTGGTCCACAGGACTTTA
USP32	GGATTGTGATGACAGCATGG	TCCCAATCCACAGCAATACA
USP33	ATTCGGCTCAACAGAGCATT	CTCCTTGCTTGAGCATCACA
USP36	CCCTGTCATGTATGGGCTCT	CCTGGAATCCGCAGATAGAA
USP37	CTGCAGGGATTCTCCAATTT	AGGTTTGCAAAGCGTCTGAT
USP38	CTGCTGGACAGACTCCATGA	CCGTAGTTTCCCTCCAAACA
USP45	TGTCAGAAGGCTCCTCGATT	TCATCTTCACGCCTTCCTTT
USP46	AGGTAGGGCTGTCATTGGTG	CCTGAAATGTGGCTTTTGGT
USP47	AGGGAGCAGTGTGGCTTAGA	CCTCCACCGTCTGGTTAAAA
USP48	AGGCGTGTGCAGAAGAAACT	ACACCTGGAGGAAGGTGTTG
CYLD	TGATCGAGATGGTGGTCAGA	GCTCATGGTTGGACTCTGGT
GAPDH	TGCTGGTGCTGAGTATGTCG	GCATGTCAGATCCACAACGG
