# Supplementary material for: Correction: Correction: Regulation of Neuronal Morphogenesis and Positioning by Ubiquitin-Specific Proteases in the Cerebellum
Source: PLoS One. 2015 Aug 5;10(8):e0135535. doi: 10.1371/journal.pone.0135535 (PMC4526667; doi:10.1371/journal.pone.0135535)
Supplement: S1 File — (PDF) [file pone.0135535.s001.pdf]

PLOS | ONE

CORRECTION

Correction: Regulation of Neuronal Morphogenesis and Positioning by Ubiquitin-Specific Proteases in the Cerebellum

Julius Anckar, Azad Bonni

There is information missing from funding section of this paper. The funding section should read as follows: The work was supported by NIH grant NS051255 (A.B.) and grants from the Swedish Cultural Foundation in Finland (J.A.) and Magnus Ehrnrooth Foundation (J.A.).

Reference

1. Anckar J, Bonni A (2015) Regulation of Neuronal Morphogenesis and Positioning by Ubiquitin-Specific Proteases in the Cerebellum. PLoS ONE 10(1): e0117076. doi: 10.1371/journal.pone.0117076 PMID: 25607801

CrossMark
click for updates

OPEN ACCESS

Citation: Anckar J, Bonni A (2015) Correction: Regulation of Neuronal Morphogenesis and Positioning by Ubiquitin-Specific Proteases in the Cerebellum. PLoS ONE 10(7): e0133943. doi:10.1371/journal.pone.0133943

Published: July 20, 2015

Copyright: © 2015 Anckar, Bonni. This is an open access article distributed under the terms of the Creative Commons Attribution License, which permits unrestricted use, distribution, and reproduction in any medium, provided the original author and source are credited.
