# Supplementary material for: Correction: Correction: Regulation of Neuronal Morphogenesis and Positioning by Ubiquitin-Specific Proteases in the Cerebellum
Source: PLoS One. 2015 Aug 5;10(8):e0135535. doi: 10.1371/journal.pone.0135535 (PMC4526667; doi:10.1371/journal.pone.0135535)
Supplement: S2 File — (PDF) [file pone.0135535.s002.pdf]

CORRECTION

Correction: Regulation of Neuronal Morphogenesis and Positioning by Ubiquitin-Specific Proteases in the Cerebellum

The *PLOS ONE* Staff

Due to a typesetting error, the Funding section is missing in this article. The Funding section should read as follows: "The work was supported by NIH grant NS051255 (A.B.) and grants from the Swedish Cultural Foundation in Finland (J.A.) and Magnus Ehrnrooth Foundation (J. A.)." The publisher apologizes for this error.

Reference

1. Anckar J, Bonni A (2015) Regulation of Neuronal Morphogenesis and Positioning by Ubiquitin-Specific Proteases in the Cerebellum. PLoS ONE 10(1): e0117076. doi: 10.1371/journal.pone.0117076 PMID: 25607801

CrossMark
click for updates

OPEN ACCESS

Citation: The *PLOS ONE* Staff (2015) Correction: Regulation of Neuronal Morphogenesis and Positioning by Ubiquitin-Specific Proteases in the Cerebellum. PLoS ONE 10(7): e0133943. doi:10.1371/journal.pone.0133943

Published: July 20, 2015

Copyright: © 2015 The PLOS ONE Staff. This is an open access article distributed under the terms of the Creative Commons Attribution License, which permits unrestricted use, distribution, and reproduction in any medium, provided the original author and source are credited.
